# Anterior Peripheral Rim Instability Is Prevalent in Young, Skeletally Immature Patients With Discoid Lateral Meniscus and Has Favorable Postoperative Outcomes

**DOI:** 10.1016/j.asmr.2025.101119

**Published:** 2025-03-25

**Authors:** Steven Maxwell Henick, Zachariah Samuel, Joseph Nicholas Charla, Emily Ferreri, Emmanuel Mbamalu, Edina Gjonbalaj, Leila Mehraban Alvandi, Jacob Foster Schulz, Eric Daniel Fornari, Mauricio Drummond

**Affiliations:** Department of Orthopaedic Surgery, Division of Pediatric Orthopaedic Surgery, Montefiore Einstein, Bronx, New York, U.S.A.

## Abstract

**Purpose:**

To report the prevalence and patient characteristics of anterior peripheral rim instability (PRI) in patients <21 years of age with symptomatic discoid lateral meniscus (DLM) requiring operative intervention and to compare preoperative magnetic resonance imaging (MRI) with arthroscopic findings and patient-reported outcomes (PROs) after arthroscopic treatment of anterior compared with nonanterior PRI.

**Methods:**

A retrospective review was performed at a single academic institution between 2012 and 2022. Patients were <21 years old and underwent operative DLM management with a minimum follow-up period of 2 years. Patients were divided into 2 groups: anterior PRI (isolated anterior PRI and anterior combined with posterior PRI) and nonanterior PRI (nonanterior PRI designated as isolated posterior or no PRI). Data collection included demographics, clinical presentation, MRI results, arthroscopic findings, reoperation rates, complications, and PROs.

**Results:**

Forty-four patients were included, 22 in the anterior PRI group and 22 in the nonanterior PRI group. Anterior PRI prevalence was 50% in this cohort. Patients in the anterior PRI group were younger (10.77 ± 3.07 vs 13.002 ± 3.39; *P* = .028) and more likely skeletally immature (16 vs 8; *P* = .034). Extension deficit (*P* = .486) did not differ significantly between the groups. MRI was less sensitive for detecting anterior PRI compared with posterior PRI (72.2% vs 95.0%; *P* = .140). Patients with anterior PRI showed significant improvements in postoperative PROs (Pedi- International Knee Documentation Committee 54.23 vs 89.65; *P* = .0006) after 6.1 years of average follow-up, achieving good-to-excellent scores that did not significantly differ between groups.

**Conclusions:**

The prevalence of anterior PRI in symptomatic DLM is 50% in our cohort and is more likely to cause symptoms in skeletally immature and younger patients. It more commonly presents with posterior PRI (59%) than as an isolated entity (41%). Arthroscopic outside-in repair yields good-to-excellent PROs and low complication and reoperation rates after mean 6.1 years of follow-up.

**Level of Evidence:**

Level III, therapeutic retrospective, cohort study.

Discoid meniscus is defined by excess meniscal surface area overlying the tibial plateau.^1^ The associated lack of meniscal-capsular attachments, and chronic strain on existing attachments, predispose to meniscal instability and tears as the result of the persistent strain of the remaining meniscal-capsular connections, resulting in peripheral rim instability (PRI), which refers to excessive meniscal mobility at the peripheral rim accompanied by a tear of the anterior and posterior horns.^1^, ^2^, ^3^ PRI should be distinguished from hypermobile meniscus, which also exhibits excessive meniscal mobility upon probing but is not necessarily accompanied by a tear. These pathologic processes likely occur as the result of collagen disorganization, especially in the posterior one third of the meniscus, and avascularity of the discoid lateral meniscus (DLM), which increase the likelihood of meniscal damage and tears.^4^, ^5^, ^6^

Literature on anterior PRI in adults with symptomatic DLM is sparse, with only one study showing increased PRI anteriorly and posteriorly in intact and torn DLM (17 knees) relative to intact lateral menisci in patients with a mean age of 44.9 ± 12.4 years.^3^ Data published from the Sports Cohort Outcomes Registry (SCORE) has previously shown a correlation between age younger than 14 years old and an increased likelihood of PRI and also complete discoid menisci leading to greater rates of meniscal repair (62.7% of patients in this age cohort); however, follow-up data or patient-reported outcomes (PROs) were not provided.^7^ In the pediatric DLM cohort of Good et al.,^8^ anterior PRI was the most common type of instability, prevalent in 53% of knees, whereas Perkins et al.^9^ note a similar incidence of anterior (44%) and posterior (50%) meniscal tears. Anterior PRI, although being very common, is also associated with female sex and subjective clicking during motion.^10^

Making an accurate diagnosis of PRI can be challenging: some studies have evaluated the accuracy of varying knee positioning on magnetic resonance imaging (MRI) for diagnosing DLM^11^ and others have focused on specific MRI findings, such as the linear fluid signal on MRI at the anterior meniscal margin or anterior parameniscal soft-tissue edema, which are associated with anterior PRI.^12^ Sensitivities and specificities for identifying a DLM tear preoperatively on MRI can range from 75% to 97.8% and 50% to 100%, respectively^1^; thus, considering risk factors for tears and instability preoperatively may assist with surgical planning and expectations.

Recent literature has also highlighted the diagnostic arthroscopic assessment and treatment of anterior PRI and anterior horn tears,^3^^,^^13^, ^14^, ^15^ which involves thorough intraoperative probing to determine whether a repair is needed despite preoperative MRI findings.^10^^,^^16^ True meniscal tears are readily detected on MRI, with high sensitivity and specificity. MRI findings such as intrasubstance signal extending to the articular surface, displaced fragments, or fluid-filled clefts allow for straightforward identification of meniscal pathology. PRI, however, is more challenging to diagnose with MRI alone. Sensitivities for detecting anterior PRI are lower than for posterior PRI, as MRI cannot directly assess meniscal mobility or meniscocapsular separation in real time. Features such as parameniscal soft-tissue edema, anterior meniscal margin signal changes, and increased meniscal extrusion may suggest PRI, but they lack the reliability of direct arthroscopic probing, which remains the gold standard for confirming instability.^17^ Furthermore, hypermobile meniscus cannot be reliably diagnosed using a single MRI scan. Unlike a true meniscal tear, where static imaging captures structural damage, hypermobile meniscus and PRI require dynamic evaluation, which MRI lacks. Weight-bearing MRI or stress imaging techniques may improve detection, but standard MRI may miss cases of instability, leading to underdiagnosis of PRI when relying solely on preoperative imaging.

Conventional classification systems, such as the Watanabe classification system, account for meniscal width and posterior peripheral instability yet fail to consider anterior instability, unlike modern classification systems (i.e., PRiSM Discoid Meniscus Classification), which account for DLM width, height, instability, and tear pattern.^14^^,^^18^ Although intra- and interobserver reliabilities of the PRiSM Discoid Meniscus Classification were lower for diagnosing an anterior compared with a posterior tear, interobserver reliability in specifying anterior and posterior PRI were both substantial.^18^

The purposes of this study are to report the prevalence and patient characteristics of anterior PRI in patients <21 years of age with symptomatic DLM requiring operative intervention and to compare preoperative MRI with arthroscopic findings and PROs after arthroscopic treatment of anterior compared with nonanterior PRI. We hypothesized that anterior PRI would have a greater prevalence than nonanterior PRI and would be more prevalent among younger female patients and that MRI findings would not always correspond to arthroscopic findings and that arthroscopic treatment of DLM with anterior and nonanterior PRI would produce adequate PRO scores.

## Methods

After receiving institutional review board approval (2020-12484), we conducted a retrospective study at a single academic institution to analyze patients who underwent arthroscopic treatment for symptomatic DLM between January 2012 and August 2022. Patients were included if they were younger than 21 years old at the time of surgery, had a minimum follow-up period of 2 years, and underwent arthroscopic treatment for symptomatic DLM. Symptomatic DLM was defined as either caused by mechanical symptoms (i.e., locking, catching, painful clicking/popping/snapping, or instability) or pain that did not resolve after at least 3 months of physical therapy. DLM was diagnosed on the basis of meniscal width of 15 mm, ratio of meniscus to the tibia of 20%, percentage coverage of the meniscus 75%, and 3 slices of continuity of the anterior and posterior horns.^19^ Patients were excluded if they did not have documented follow-up in clinic or of PROs after at least 2 years.

The cohort was divided on the basis of the presence and location of PRI: anterior PRI consisted of all patients with anterior PRI including isolated anterior PRI and combined anterior and posterior PRI. Nonanterior PRI consisted of patients without anterior PRI, including those with isolated posterior or no PRI. On the basis of the PRiSM Lateral Discoid Meniscus Classification System, anterior PRI consists of instability of only the anterior horn with or without the involvement of the midbody (anterior PRI group). Posterior PRI encompassed only the posterior horn with or without the midbody (nonanterior PRI group). Combined anterior and posterior PRI had involvement of the anterior horn, midbody, and posterior horn (anterior PRI group).^20^

Demographics including age, sex, ethnicity, and body mass index were collected. Skeletal maturity was determined via radiographs of the knee to assess physeal status according to the technique described by Dekhne et al.,^21^ with stage 4 (bony/fused) categorized as skeletally mature. Clinical presentations were reported by documenting mechanical symptoms, such as locking and catching, and preoperative restricted range of motion, specifically extension deficit, compared with the contralateral side. If the contralateral knee was also symptomatic, patients were instead asked to compare their knee function to how it felt before the onset of symptoms.

MRIs were completed with the patient in standard supine position with knee imaged in the anteroposterior plane with a standard 16-channel knee coil to obtain the following sequences: coronal T1-weighted, coronal proton density fat-saturated, sagittal proton density, sagittal proton density fat-saturated, and axial proton density fat-saturated. MRIs were read by a musculoskeletal fellowship-trained radiologist with particular attention to meniscal width, the ratio of meniscal-to-tibial width, percent coverage of the meniscus, and continuity between the anterior and posterior horns.

**Fig 1:**
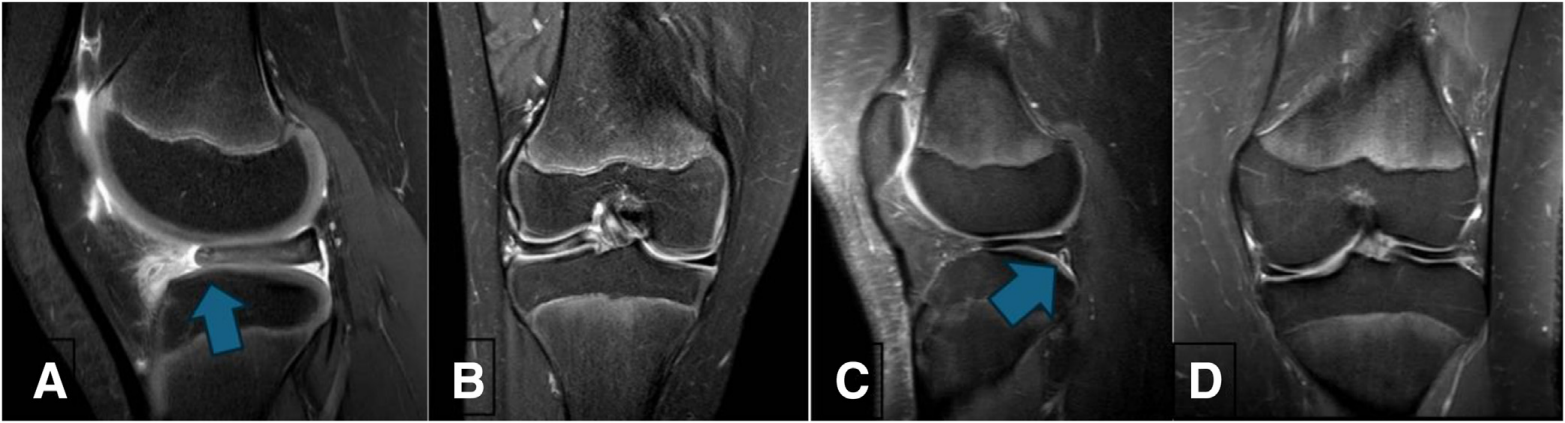
Representative sagittal (A) and coronal (B) cuts of proton-dense fat-suppressed sequences of a right knee with anterior peripheral rim instability (PRI) patient showing posterior displacement of the anterior horn of the lateral meniscus (arrow). There is resultant signal hyperintensity remains where the anterior horn should be attached. The coronal image shows the lack of visible lateral meniscus where it should be present compared with the contralateral side. An example of a posteriorly located fluid collection (arrow) in (C) and (D) is suggestive of isolated posterior instability for a patient with nonanterior PRI. All magnetic resonance imaging scans were obtained in the supine position with the knee extended.

**Fig 2:**
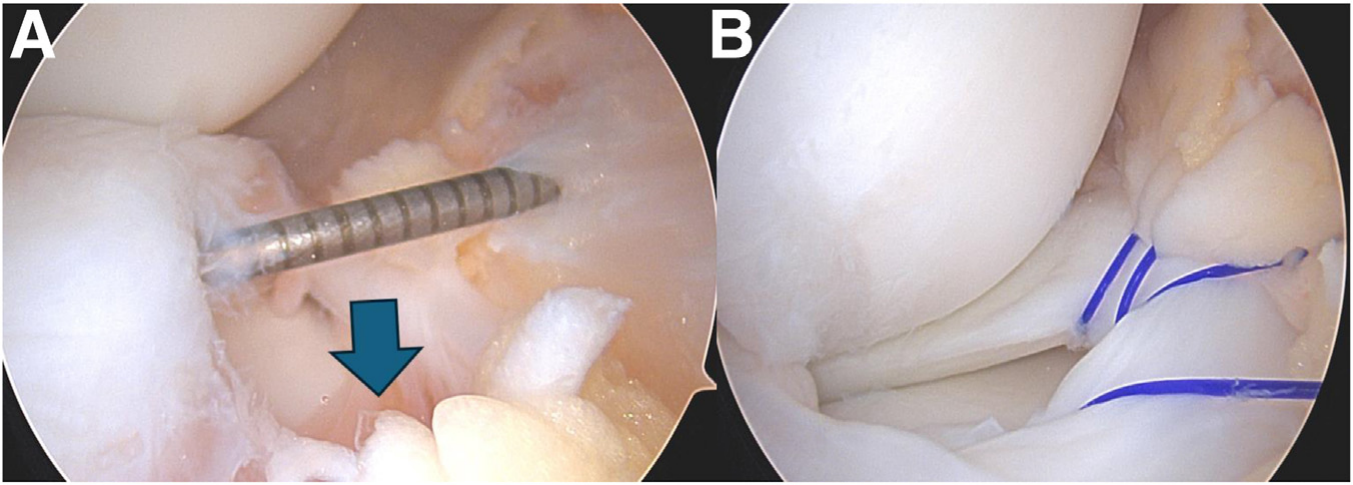
Left knee view from the anteromedial arthroscopic portal viewing the anterior lateral meniscus. (A) Probing through the anterolateral portal shows the lateral anterior horn meniscocapsular separation with resultant displacement (arrow). (B) Repair was done via an outside-in technique using 4 polydioxanone No. 0 sutures.

**Fig 3:**
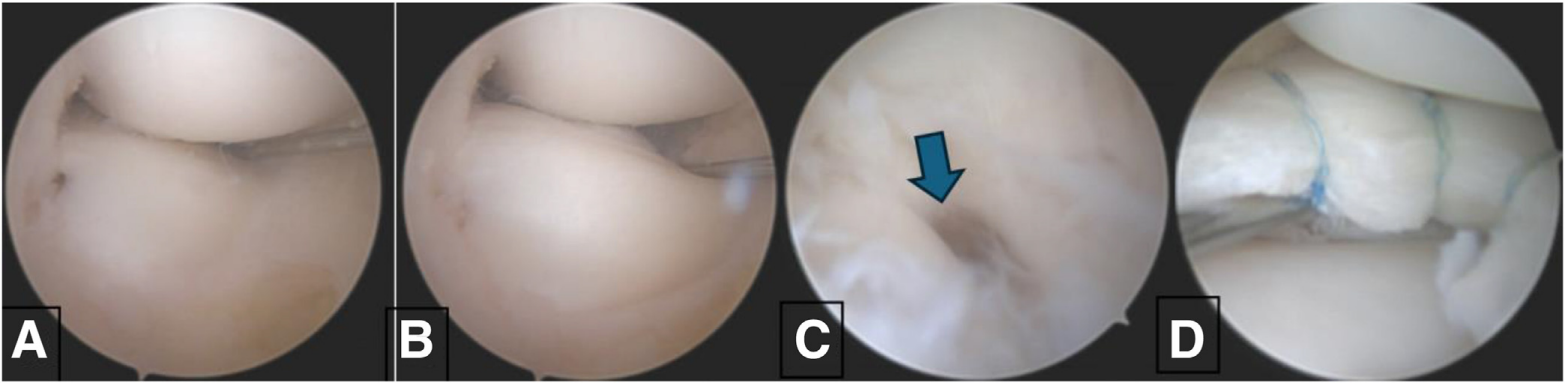
Left knee view from the anteromedial portal with probe in the anterolateral portal demonstrating a dynamic assessment of the posterior horn peripheral instability. Before (A) and after (B) provocative test. (C) Arthroscope in the anterolateral portal demonstrating a complete posterior meniscocapsular separation (arrow). (D) Demonstration of saucerization and all-inside repair with Fast-Fix.

The reports were compared with intraoperative arthroscopic findings by a medical student and verified by a fellowship-trained pediatric sports orthopaedic surgeon with more than 10 years of experience (M.D.J.). MRI findings showing evidence of anterior and nonanterior PRI are shown in Figure 1. Arthroscopic images for a patient with anterior PRI are shown in Figure 2 and arthroscopic images for a patient with nonanterior PRI are shown in Figure 3.

### Surgical Procedure

Two surgeons (J.S. and E.F.) at our institution performed arthroscopic management for patients with DLM by the use of consistent surgical steps and implants (Fast-Fix Meniscal Repair System, Smith & Nephew, Andover, MA). Diagnostic arthroscopy was performed in a standard fashion. The arthroscope was first placed in the anterolateral portal and the probe inserted in the anteromedial portal to carefully evaluate the body and posterior horn segments to identify posterior PRI and tears. The arthroscope was then moved to the anteromedial portal and the probe to the anterolateral portal to assess for meniscocapsular separation, and thus, anterior PRI. Then, saucerization was done first with the goal to achieve a meniscus rim between 8 and 10 mm. If identified intraoperatively, posterior PRI was addressed with an all-inside technique (Fast-Fix Flex Meniscal Repair System) with the arthroscope in the anterolateral portal while the suture device was inserted through the anteromedial portal to minimize risk of iatrogenic neurovascular injury. Preoperative planning included analysis of the axial cut of MRI to guide safe meniscal repair technique as described by previous literature.^22^ For patients with an anterocentral shift of the discoid lateral meniscus—where the peripheral rim of the posterior horn is particularly small—the risk of toggle and subsequent anchor pull-out is heightened. To mitigate this, the Fast-Fix was adjusted to the longest setting (18 mm) and the first implant was carefully placed within 3 to 4 mm (and, in cases of vertical tears, no more than 5 mm from the tear) to transfix the meniscus to the capsule. The second implant was then positioned directly through the posterior capsule in a vertical mattress fashion. For horizontal cleavage tears, the Fast-Fix was inserted directly through the capsule above and below the meniscus, again in a vertical mattress configuration.

Anterior PRI was stabilized with an outside-in technique without implants as described by Chahla et al.^23^ Saucerization was first performed with the goal to achieve a meniscus rim between 8 and 10 mm. We then used a No. 0 PDS suture (Ethicon, a Johnson & Johnson Company, Somerville, NJ), which was passed through the needle, followed by a second needle inserted in the anterior capsule at a more superior position as a suture retriever with a loop. The loop was fed with a grasper to capture the suture, which was subsequently shuttled outside the knee. To enhance meniscal healing and stability, a “hay-baling” technique was used, mirroring approaches used for horizontal meniscus tears. This involved placing multiple evenly spaced sutures (3-5 mm apart) perpendicular to the meniscocapsular separation, effectively closing the gap between the meniscus and capsule while distributing tension evenly across the repair site. This reduces the risk of suture pull-through and prevents tear propagation while allowing the meniscus to heal under more uniform pressure. A small 1-cm incision was made in front of the knee, and after blunt dissection to clear the soft tissue, the suture was tied over the anterior aspect of the anterior capsule, securing the repair.

To enhance healing, we first used meniscal rasp in order to remove scar tissue and at the end of the procedure, we used bone marrow venting on the lateral femoral condylar notch with an ice pick to promote fibrochondral integration at the menisculocapsular junction.

Study grouping was determined on the basis of arthroscopic findings. All meniscal repairs followed the same rehabilitation protocol including weight-bearing as tolerated with a knee brace locked in extension and early passive range of motion for the first 6 weeks. After 6 weeks, the brace was discontinued, and the patient continued muscle strengthening and proprioception exercises. Squatting is permitted after 3 months, starting gradually from 0 to 45°, 60°, then 90° and greater.

Postoperative data were collected regarding the need for repeat surgery and complications. PRO scores were obtained starting at the 1-year follow-up appointment either during office visits or via telephone calls using validated instruments measuring knee function and patient satisfaction.^24^, ^25^, ^26^ The PROs collected included the Tegner Lysholm score, Pediatric International Knee Documentation Committee (Pedi-IKDC) subjective knee evaluation form, and Knee injury and Osteoarthritis Outcome Score for Children (KOOS-Child). Greater scores indicate better outcomes across all PRO measures, and minimal clinically important difference was determined on the basis of previous literature.^27^ The content of both questionnaires remains the same, as KOOS-Child merely rephrases the questions to be more comprehensible for children.^28^ All patients were contacted at the final follow-up to inquire about their functional status, including returning to sports, activities of daily living, and satisfaction with the surgical outcome. Patients who lacked postoperative PROs or reported that they had not returned to their preoperative activity level by the final in-person follow-up were called via telephone and asked additional questions regarding their functional status and satisfaction with their operative knee. All questions were asked as they are printed in Table 1 to ensure standardization across all patients who were contacted. Specifically, knee function was asked in comparison with the contralateral unaffected knee similar in the way that the Single Assessment Numeric Evaluation score is calculated in patients with shoulder pathologies.^29^

**Table 1:** Qualitative Analysis of Patient Responses if They Had Incomplete PRO Data on Return to Activities

Patient No.	Group	Do You Have Pain or Mechanical Symptoms in the Operated Knee?	Did You Have Any Additional Surgery in the Operated Knee?	Were You Able to Return to Activities? Sports? What Type?	How Is Your Operated Knee Function Compared With the Nonoperated Knee From 0% (Worst) to 100% (Completely Normal)?	How Are You Satisfied With Your Surgery Result From 0% to 100%?
1	Nonanterior PRI	No	No	Yes, basketball	85%	95%
2	Nonanterior PRI	No	No	Yes, basketball	100%	100%
3	Anterior PRI	No	No	Yes, basketball	96%-97%	100%
4	Nonanterior PRI	Yes	No	No, football and basketball	80%	50%

PRI, peripheral rim instability; PRO, patient reported outcome.

### Statistical Analysis

Data were presented as means and standard deviations or medians and interquartile ranges for continuous variables. Frequencies and percentages were done for categorical variables. Demographic differences and location of instability of the patient’s discoid meniscus were assessed using *t* tests or Wilcoxon rank-sum tests for continuous variables and χ^2^ or Fisher exact tests for categorical variables. Wilcoxon rank-sum tests compared the postoperative PRO scores between patients with anterior and nonanterior PRI. Wilcoxon matched-pairs signed rank test was used to evaluate the difference in the cohort’s preoperative PROs and postoperative PROs. Statistical analyses were performed using STATA software, version 18 (STATA Corp, College Station, TX) and R (version 4.2.1; R Foundation for Statistical Computing, Vienna, Austria). *P* values of .05 or less were considered statistically significant. An alpha level of .05, χ^2^ statistic of 59, 3 degrees of freedom, and an effect size of 1.0 were used. With a beta of 0.0 (power = 1.0), the test had sufficient power to detect differences between the groups.

## Results

**Fig 4:**
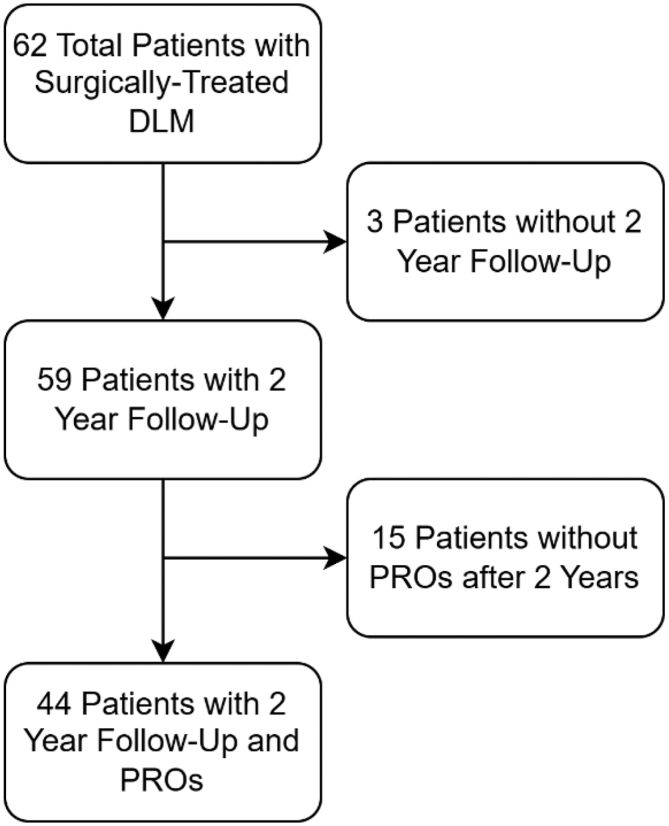
Patient flowchart detailing number of patients with 2 year follow up and PROs. (DLM, discoid lateral meniscus; PRO, patient-report outcome.)

There were 44 patients (mean age 11.89 ± 3.39 years, range 6-20 years) in the initial cohort who underwent surgical management; all met inclusion criteria (Fig 4). Of these, 22 presented with anterior PRI (either isolated or combined with posterior PRI) and the other 22 presented with nonanterior PRI (isolated posterior or no PRI). Overall, the prevalence of anterior PRI in our cohort was 50.0%.

Patients with anterior PRI were younger (10.77 ± 3.07 vs 13.00 ± 3.39, *P* = .028) and primarily skeletally immature (16 vs 8, P = .034), with the oldest skeletally immature patient being 16 years old. No significant differences were observed in ethnicity (*P* = .857), body mass index (*P* = .223), mechanical symptoms (*P* = .130), or extension deficit (*P* = .486) (Table 2). Mean number of Fast-Fix used during surgery was 4 ± 1.39. Although not statistically significant, MRI was less sensitive for detecting anterior PRI compared with posterior PRI (72.2% vs 95.0 %, *P* = .140).

**Table 2:** Demographics and Characteristics of all Patients Separated on the Basis of the Presence of Anterior PRI Versus Nonanterior PRI

Demographics and Characteristics of the Participants n = 44		Location of Instability		*P* Value
		Anterior PRI	Nonanterior PRI	
		n = 22	n = 22	
Age, yr, mean ± SD	11.89 ± 3.39	10.77 ± 3.07	13.00 ± 3.39	.028
Sex, no. (%)				.227
Male	23 (52.3)	9 (40.90)	14 (63.6)	
Female	21 (47.7)	13 (59.09)	8 (36.4)	
Ethnicity n = 39, no. (%)				.857
Black	6 (15.4)	3 (15.0)	3 (15.8)	
Hispanic	30 (76.9)	15 (75.0)	15 (78.9)	
White	3 (7.7)	2 (10.0)	1 (5.3)	
BMI	24.52 ± 5.58	23.48 ± 5.06	25.55 ± 5.99	.223
Skeletal maturity				.034
Immature	24 (54.5)	16 (72.73)	8 (36.4)	
Mature	20 (45.5)	6 (27.30)	14 (63.6)	
Mechanical symptoms (i.e., locking, catching, painful clicking/popping/snapping, or instability)				.130
Yes	25 (59.5)	9 (45.00)	16 (72.7)	
No	17 (40.5)	13 (59.09)	6 (27.3)	
Extension deficit				.486
Yes	11 (25.0)	7 (31.82)	4 (18.2)	
No	33 (75.0)	15 (68.2)	18 (81.8)	
MRI consistent with arthroscopic findings n = 38				.140
Yes	32 (84.2)	13 (72.2)	19 (95.0)	
No	6 (15.8)	5 (27.80)	1 (5.0)	
Procedure				<.001
Saucerization	13 (29.5)	0 (0.00)	13 (59.1)	
Posterior repair	9 (20.5)	0 (0.00)	9 (40.9)	
Anterior repair	9 (20.5)	9 (40.9)	0 (0.00)	
Anterior and posterior repair	13 (29.5)	13 (59.1)	0 (0.00)	
Complications/reoperations				>.99
Yes	3 (6.8)	1 (4.5)	2 (9.1)	
No	41 (93.2)	21 (95.5)	20 (90.9)	
Return to activity				>.99
Yes	43 (97.7)	22 (100)	21 (95.4)	
No	1 (2.3)	0 (0)	1 (4.6)	

BMI, Body mass index; MRI, magnetic resonance imaging; PRI, peripheral rim instability; SD, standard deviation.

All 22 patients with anterior PRI underwent saucerization and meniscal repair: 9 (40.90%) underwent isolated anterior repair, and 13 (59.10%) underwent combined anterior and posterior repair. Among the 22 patients with nonanterior PRI, 13 (59.10%) were stable and underwent saucerization alone, whereas the remaining 9 (40.90%) had a posterior PRI repair.

Collectively across groups, there were statistically significant increases in all preoperative PROs (Tegner 53.33 vs 92.50, *P* = .03; Pedi-IKDC 53.52 vs 89.65, *P* < .01; KOOS-symptom 60.70 vs 89.29, *P* < .01; KOOS-pain 50.00 vs 93.30, *P* < .01; KOOS-ADL 63.64 vs 97.87, *P* < .01; KOOS-sport 46.43 vs 92.86, *P* < .01; KOOS-QOL 41.67 vs 79.17, *P* < .01)at an average of 6.05 years follow-up (Table 3). Minimal clinically important difference was reached in a large proportion of cases for each PRO: Tegner-Lysholm (62.5%), Pedi-IKDC (85.7%), KOOS-symptom (78.6%), KOOS-pain (85.7%), KOOS-ADL (64.3%), KOOS-sport (61.5%), and KOOS-QOL (50.0%).

**Table 3:** Comparison of Preoperative and Postoperative PROs Across the Entire Cohort

PROs	Preoperative and n = 16	Postoperative n = 44	MCID	*P* Value
Tegner Lysholm	53.33 [40.50-76.50]	92.50 [81.25-100.00]	62.5%	.03
Pedi-IKDC	53.52 [37.64-60.92]	89.65 [68.46-95.55]	85.7%	<.01
KOOS Child Symptom	60.70 [44.00-75.89]	89.29 [75.00-100.00]	78.6%	<.01
KOOS Child Pain	50.00 [40.25-70.31]	93.30 [83.59- 100.00]	85.7%	<.01
KOOS Child ADL	63.64 [55.91- 84.43]	97.87 [88.63-100.00]	64.3%	<.01
KOOS Child Sport	46.43 [32.25-58.39]	92.86 [78.57-100.00]	61.5%	<.01
KOOS Child QOL	41.67 [31.25-68.33]	79.17 [68.75-87.50]	50.0%	<.01

ADL, activities of daily living; KOOS, Knee Injury and Osteoarthritis Outcome Score; MCID, minimal clinically important difference; Pedi-IKDC, Pediatric International Knee Documentation Committee; PROs, patient reported outcome; QOL, quality of life.

There were no statistically significant differences in the most recently obtained postoperative PROs at an average of 6.05 years follow-up between the anterior and nonanterior PRI groups in the Tegner Lysholm score (95.50 vs 86.00, *P* = .34), Pedi-IKDC (93.10 vs 87.00, *P* = .28), KOOS-symptom (91.67 vs 82.14, *P* = .46), KOOS-pain (96.88 vs 87.50, *P* = .19), KOOS-ADL (100.00 vs 95.45, *P* = .12), KOOS-sport (94.65 vs 92.86, *P* = .38), and KOOS-QOL (79.16 vs 79.17, *P* = .37) (Table 4).

**Table 4:** Comparison of Postoperative PROs Between Patients with Anterior PRI and Nonanterior PRI

Postoperative PROs	Location of Instability		*P* Value
	Anterior PRI n = 21	Nonanterior PRI n = 20	
Tegner Lysholm	95.50 [84.75-100.00]	86.00 [78.75-96.75]	.34
Pedi-IKDC	93.10 [70.11-96.55]	87.00 [61.40-94.83]	.28
KOOS Child Symptom	91.67 [85.71-100.00]	82.14 [69.43-100.00]	.46
KOOS Child Pain	96.88 [87.50-100.00]	87.50 [79.69-98.44]	.19
KOOS Child ADL	100.00 [93.18-100.00]	95.45 [85.23-100.00]	.12
KOOS Child Sport	94.65 [85.71-100.00]	92.86 [66.07-100.00]	.38
KOOS Child QOL	79.16 [70.83-89.96]	79.17 [45.83-83.33]	.37

ADL, activities of daily living; KOOS, Knee Injury and Osteoarthritis Outcome Score; Pedi-IKDC, Pediatric International Knee Documentation Committee; PRI, peripheral rim instability; PROs, patient reported outcome; QOL, quality of life.

Although an extensive chart review confirmed that almost all patients (39/44, 88.6%) were ultimately cleared to return to activities, 4 of 44 patients (9.1%) did not complete postoperative PROs.^29^ From these 4 patients who were able to be reached and who answered a follow-up questionnaire, only 1 patient was not able to return to their preinjury status (patient 4). All other patients reported being able to return to their preinjury status with 95% to 100% satisfaction.

On the basis of an average follow-up of 6.05 ± 2.36 (2.37-11.50) years, there was no statistically significant difference between groups regarding complications and reoperations (1 anterior vs 2 nonanterior, *P* > .99). Of the 44 patients, 3 experienced long-term complications with 2 requiring a second surgical intervention (knee arthroscopy). There were 2 meniscus retears: 1 among the patients with anterior PRI and 1 among the patients with nonanterior PRIs. Both patients had a history of new trauma and remembered the event preceding the new tear. The patient with anterior PRI had knee pain and swelling triggered when rising from sitting that occurred spontaneously 427 days after the initial operation. The patient with nonanterior PRI fell on ice 517 days after the initial procedure. One patient with previous meniscus repair had the original tear on the anterior horn. Repeat knee arthroscopy showed that the anterior horn tear was fully healed and there was a new horizontal cleavage on the body of the lateral meniscus that was trimmed. There was one case of arthrofibrosis and this patient improved with non-operative treatment. No neurovascular complications were reported.

## Discussion

The most important finding of this study was that the prevalence of DLM with anterior PRI in this cohort is 50%. Anterior PRI presents more commonly in association with posterior PRI (59%) than as an isolated entity (41%). Anterior PRI is more likely to be symptomatic in younger, skeletally immature patients. Our data add to the growing literature of symptomatic DLM treated surgically and encourages surgeons to comprehensively assess each patient for an anterior meniscocapsular separation arthroscopically even if this is not visible on preoperative MRI. At an average 6.1 years of follow-up, PROs after arthroscopic anterior PRI repair are good-to-excellent with low complication and revision rates similar to isolated posterior or no PRI patients.

In 2004, Klingele et al.^16^ reviewed outcomes of operatively managed symptomatic DLM in pediatric patients: a review of 128 knees in patients at an average 10.0 years of age revealed a PRI rate of 28.1%. Of this cohort, 47.2% patients had instability in the anterior third of the DLM, which was greater than the posterior third (38.9%) or peripheral middle third (11.1%) cohorts. Good et al.^8^ also reported anterior horn instability in the majority of their 30-knee cohort (53%), which they addressed with outside-in repair. Their cohort achieved knee flexion greater than 135° after 3 years of follow-up. More recently, Perkins et al.^9^ evaluated the outcomes of patients with DLM who underwent saucerization and meniscal repair in the setting of known PRI. In 32 knees with patients of a mean age 12 years (5-17 years), there were 14 (44%) anterior and 16 (50%) posterior meniscocapsular tears (6% had both anterior and posterior tears). These studies all suggest that our rate of 50.0% (22/44) patients with anterior instability in our cohort is reasonable and consistent with previous literature.

In our cohort, younger age (10.77 ± 3.07) and skeletal immaturity were associated with anterior PRI while older age (13.00 ± 3.39) and skeletal maturity were more closely associated with posterior or no PRI. The association between younger age and PRI has been illustrated previously by Klingele et al.,^16^ who showed that younger individuals, which they constituted as 8.2 versus 10.7 years of age, had greater risks of PRI. However, more recently this has been disputed: a retrospective review of 78 knees at a mean 9.9 years of age found that of the 40 knees with PRI, they had an average 9.8 years of age versus 10.1 years old in the cohort without PRI which did not meet statistical significance.^30^ In a larger, multicenter study, authors from the SCORE database documented that in patients younger than 10 years old there was a statistically significant increase in the number of patients having concomitant PRI compared with patients older than 16 years old. Interestingly, the younger patients also had greater rates of meniscal repair compared with the oldest ones. Although their cohort’s average age was 12.4 years, ours at a younger age of 11.9 years may explain the finding of anterior or posterior PRI among all the patients in our cohort. Furthermore, although we found an association between younger age and anterior PRI with a greater rate of posterior PRI with increasing age, the opposite was true in the SCORE study.^7^ Simon et al.^31^ suggested the high instability rate (94%) in their cohort of 114 knees might be due to their average age of 11 years.^32^^,^^33^

Physicians may increase the preoperative likelihood of diagnosing anterior PRI by appreciating clicking during knee range of motion as prompt recognition is crucial to prevent worsening meniscal tears.^10^^,^^31^ Snapping knee may indicate anterior PRI even if the DLM appears stable on MRI,^11^ whereas extension deficits over 10° are more commonly linked to middle body or posterior PRI.^10^^,^^34^ Thorough knee flexion examination compared with the contralateral leg may reveal abnormalities when extension deficits are absent.^31^ Ahn et al.^35^ in 2009 noted that patients experience symptoms more commonly when there is a tear either anteriorly or posteriorly, and the authors note that patients with meniscal shift on MRI were more commonly to be diagnosed with a tear preoperatively to which they emphasize the importance of a thorough preoperative physical examination to substantiate or potentially call into question the MRI findings. In our cohort, mechanical symptoms and extension deficits did not differ significantly between groups which highlights the importance of diagnostic arthroscopy due to physical examination limitations.

Even though our data did not reach statistical significance, there was a trend to support MRI being less sensitive to detecting anterior PRI. Knee positioning affects where DLM appears on MRI: extension, the DLM that is unstable anteriorly will displace posteriorly in extension and vice versa when the knee is flexed.^10^ In addition, assessing for a linear fluid signal and parameniscal soft-tissue edema at the anterior meniscal margin have a high specificity and positive predictive value in diagnosing anterior PRI in the context of no-shift complete DLM.^12^

Compared with 5.0% for posterior PRI, 27.8% of anterior PRI diagnoses made arthroscopically did not correspond with MRI findings. All patients in this study underwent supine MRIs under standard protocol, but published literature on weight-bearing MRI notes a decrease in the space between the distal aspect of the femoral condyle and surface of the tibial plateau that may provide more accurate diagnostic capabilities.^36^ In addition, when giving close attention to no-shift DLM intraoperatively to assess for potential instability,^35^ the finding of a no-shift complete DLM can be associated with PRI specifically anteriorly. In both adult and pediatric patients with a preoperatively documented snapping knee on flexion and terminal extension on physical examination, a 2-positioned MRI for these patients found a rate of PRI of 71.8% patients (average 14.6 years old) had anteriorly-based instability.^11^ In a retrospective review of MRIs of 79 of weight-bearing knees, the authors found that PRI presented as a shift within the intercondylar notch with resultant extension deficit and increased likelihood of having associated clicking, clunking, and limited extension on examination; however, these results are attributable to PRI more broadly and not into specific locations of instability.^36^ Recent research highlights the phantom sign’s near perfect intrarater and substantial interrater reliability with high sensitivity (95%) for detecting anterior instability in lateral menisci. Other MRI findings, such as lateral extrusion, anterior horn signal, and posterior megahorn, also can indicate instability. However, MRI has limitations in assessing PRI in DLM patients, underscoring the need for thorough diagnostic arthroscopy regardless of MRI findings.^32^

Arthroscopic techniques have evolved to improve diagnosis and treatment of anterior PRI^13^, ^14^, ^15^^,^^37^ considering more than 25% of diagnoses are overlooked on preoperative MRI. Authors recommend anteromedial and anterolateral portals^13^^,^^14^ for visualization with the anteromedial portal effective for visualizing anterior horn tears after shaving overlying connective tissue and extending the knee.^13^^,^^14^ Probing through both portals can reveal PRI hidden by synovial tissue,^9^ whereas a far medial accessory portal can better visualize the anterior meniscus.^37^ Repairs are often performed anteriorly via outside-in suturing^12^^,^^38^ with 3- to 4-mm spacing between sutures.^13^^,^^38^ After anterior repair, the posterior meniscus should be assessed with a probe and repaired if it shifts anteriorly into the femoral notch.^13^

Perkins et al.^9^ previously showed that in their cohort of 32 knees that 3 ultimately underwent revision surgery but that all these patients previously underwent repair of the posterior as opposed to anterior horn. The mean final follow-up IKDC score was 96 (82-100), and 89% had returned at least to the same pre-operative level of activity.^9^ Another study reported an 81.6% effectiveness rate in their cohort of 57 patients with improvement in IKDC score by 35 points compared with preoperatively.^38^ Most recently, similar to our findings, Talathi et al.^39^ reported in their retrospective review of 41 pediatric patients with DLM that there were no differences between their anterior and nonanterior meniscal horn tear patients at 2 years postoperatively on the Pedi-IKDC or the Patient Assessment Questionnaire. On the basis of our cohort, surgeons can expect substantial post-operative improvements in PROs, achieving scores in the good-to-excellent range with a low complication rate of 6.8%, including 2 retears and 1 patient with arthrofibrosis. These significant improvements in PROs combined with a low complication rate makes this an effective and safe treatment for DLM with anterior PRI. Results were similarly favorable for our nonanterior PRI cohort which is consistent with previous literature.

## Limitations

This study has several limitations. First, an a priori power analysis was not done for this study, so it may be under powered. Not all patients completed postoperative PROs possibly as a result of the length of the assessments; fewer questionnaires may have improved response rates. The rarity of this condition and its surgical treatment limited the availability of long-term outcome measures. Standard supine MRI positioning may have reduced accuracy in detecting anterior PRI pre-operatively. Small subgroup sizes, such as isolated posterior PRI and no PRI in the nonanterior PRI group, required combining patients into 2 larger groups which could have potentially masked the specific effects of posterior or no PRI in other parts of the meniscus aside from only anteriorly. In addition, given the incidence of bilateral DLM, some patients may have had contralateral pathology, even if asymptomatic, which was not accounted for in this analysis. Objective evaluation using follow-up MRI or routine second-look arthroscopy to assess meniscal healing was not performed, limiting direct assessment of structural outcomes. Furthermore, preoperative PROs were available for only 22 patients, potentially limiting baseline comparisons. Finally, we used Pedi-IKDC and KOOS-Child in older subjects. The Pedi-IKDC is designed for up to age 18 years, and the KOOS-Child is designed for use up to age 17 years. We have patients who were up to 21 years of age at the time of surgery and were followed for 2 to 11 years. The reliability of these pediatric measures for adults is unknown. However, the content of both questionnaires remains the same, as KOOS-Child merely rephrases the questions to be more comprehensible for children.^28^ It is reasonable to assume that if these pediatric versions are designed to be easily understood by children, older patients should also be able to accurately interpret and respond to the questions, yielding comparable results to the adult versions of these instruments.

## Conclusions

The prevalence of anterior PRI in symptomatic DLM is 50% in our cohort and is more likely to cause symptoms in skeletally immature and younger patients. It more commonly presents in combination with posterior PRI (59%) as opposed to as an isolated entity (41%). Arthroscopic outside-in repair yields good-to-excellent PROs and low complication and reoperation rates after mean 6.1 years of follow-up.

## Disclosures

All authors (S.M.H., Z.S., J.N.C., E.F., E.M., E.G., L.M.A., J.F.S., E.D.F., M.D.) declare that they have no known competing financial interests or personal relationships that could have appeared to influence the work reported in this paper.

## References

[1] Niu E.L., Lee R.J., Joughin E., Finlayson C.J., Heyworth B.E. (2022). Discoid meniscus. Clin Sports Med.

[2] Kocher M.S., Logan C.A., Kramer D.E. (2017). Discoid lateral meniscus in children: Diagnosis, management, and outcomes. J Am Acad Orthop Surg.

[3] Kim J.H., Bin S.I., Lee B.S., Kim J.M., Kim N.K., Lee C.R. (2018). Does discoid lateral meniscus have inborn peripheral rim instability? Comparison between intact discoid lateral meniscus and normal lateral meniscus. Arch Orthop Trauma Surg.

[4] Tudisco C., Botti F., Bisicchia S. (2019). Histological study of discoid lateral meniscus in children and adolescents: Morphogenetic considerations. Joints.

[5] Bisicchia S., Botti F., Tudisco C. (2018). Discoid lateral meniscus in children and adolescents: A histological study. J Exp Orthop.

[6] Pace J.L., Luczak S.B., Kanski G., Fitzsimmons K.P., Kakazu R. (2021). Discoid lateral meniscus saucerization and treatment of intrasubstance degeneration through an accessory medial portal using a small arthroscope. Arthrosc Tech.

[7] Silverstein R.S., McKay S.D., Coello P. (2023). Relationship between age and pathology with treatment of pediatric and adolescent discoid lateral meniscus: A report from the SCORE Multicenter Database. Am J Sports Med.

[8] Good C.R., Green D.W., Griffith M.H., Valen A.W., Widmann R.F., Rodeo S.A. (2007). Arthroscopic treatment of symptomatic discoid meniscus in children: Classification, technique, and results. Arthroscopy.

[9] Perkins C.A., Busch M.T., Christino M.A., Willimon S.C. (2021). Saucerization and repair of discoid lateral menisci with peripheral rim instability: Intermediate-term outcomes in children and adolescents. J Pediatr Orthop.

[10] Kang M.S., Kim J.M., Park S.S., Bin S.I. (2019). Prediction of the peripheral rim instability of the discoid lateral meniscus in children by using preoperative clinicoradiological factors. J Pediatr Orthop.

[11] Hashimoto Y., Nishino K., Yamasaki S., Nishida Y., Takahashi S., Nakamura H. (2022). Two positioned MRI can visualize and detect the location of peripheral rim instability with snapping knee in the no-shift-type of complete discoid lateral meniscus. Arch Orthop Trauma Surg.

[12] Hashimoto Y., Nishino K., Yamasaki S., Nishida Y., Takahashi S., Nakamura H. (2021). Predictive signs of peripheral rim instability with magnetic resonance imaging in no-shift-type complete discoid lateral meniscus. Skeletal Radiol.

[13] Hashimoto Y., Yamasaki S., Guttmann D. (2022). Surgical management of discoid lateral meniscus with anterior peripheral instability: Retaining an adequate residual meniscus volume. Arthrosc Tech.

[14] Beck J.J., Schlechter J., Schmale G., Haus B., Lee J. (2022). Comprehensive arthroscopic characterization of discoid meniscus tears and instability using the PRiSM discoid meniscus classification. Arthrosc Tech.

[15] Suzuki T., Matsumura T., Otsubo H., Kuroda M. (2021). Meniscus repair with anterior cord release for peripheral tear type of discoid lateral meniscus. Arthrosc Tech.

[16] Klingele K.E., Kocher M.S., Hresko M.T., Gerbino P., Micheli L.J. (2004). Discoid lateral meniscus: Prevalence of peripheral rim instability. J Pediatr Orthop.

[17] Restrepo R., Weisberg M.D., Pevsner R., Swirsky S., Lee E.Y. (2019). Discoid meniscus in the pediatric population: Emphasis on MR imaging signs of instability. Magn Reson Imaging Clin N Am.

[18] Niu E.L., Milewski M.D., Finlayson C.J. (2023). Reliability of MRI interpretation of discoid lateral meniscus: A multicenter study. Orthop J Sports Med.

[19] Samoto N., Kozuma M., Tokuhisa T., Kobayashi K. (2002). Diagnosis of discoid lateral meniscus of the knee on MR imaging. Magn Reson Imaging.

[20] Lee R.J., Nepple J.J., Schmale G.A. (2022). Reliability of a new arthroscopic discoid lateral meniscus classification system: A multicenter video analysis. Am J Sports Med.

[21] Dekhne M.S., Kocher I.D., Hussain Z.B. (2021). Tibial tubercle apophyseal stage to determine skeletal age in pediatric patients undergoing ACL reconstruction: A validation and reliability study. Orthop J Sports Med.

[22] Beck J.J., Shifflett K., Greig D., Ebramzadeh E., Bowen R.E. (2019). Defining a safe zone for all-inside lateral meniscal repairs in pediatric patients: A magnetic resonance imaging study. Arthroscopy.

[23] Chahla J., Gannon J., Moatshe G., LaPrade R., LaPrade R.F., Arendt E.A., Getgood A., Faucett S.C. (2017). The menisci.

[24] van der Velden C.A., van der Steen M.C., Leenders J., van Douveren F., Janssen R.P.A., Reijman M. (2019). Pedi-IKDC or KOOS-child: Which questionnaire should be used in children with knee disorders?. BMC Musculoskelet Disord.

[25] Chamorro-Moriana G., Perez-Cabezas V., Espuny-Ruiz F., Torres-Enamorado D., Ridao-Fernández C. (2022). Assessing knee functionality: Systematic review of validated outcome measures. Ann Phys Rehabil Med.

[26] Martinkėnienė V.B., Austys D., Šaikus A. (2023). The significance of selecting an appropriate patient-reported outcome measure (PROM): A cross-cultural adaptation of the specific Paediatric International Documentation Committee Subjective (Pedi-IKDC) Knee Form. Children (Basel).

[27] Qiao Y., Wu C., Wu X. (2024). The value of minimal clinically important difference, substantial clinical benefit, and patient-acceptable symptomatic state for commonly used patient-reported outcomes in recurrent patellar instability patients after medial patellofemoral ligament reconstruction and tibial tubercle transfer. Arthroscopy.

[28] Örtqvist M., Roos E.M., Broström E.W., Janarv P.M., Iversen M.D. (2012). Development of the Knee Injury and Osteoarthritis Outcome Score for children (KOOS-Child): Comprehensibility and content validity. Acta Orthop.

[29] Nazari G., MacDermid J.C., Bobos P., Furtado R. (2020). Psychometric properties of the Single Assessment Numeric Evaluation (SANE) in patients with shoulder conditions. A systematic review. Physiotherapy.

[30] Ammann N., Kaelin R., Ammann E. (2023). Meniscal rim instability has a high prevalence and a variable location. Arch Orthop Trauma Surg.

[31] Simon V., Paul Henri B., Charles F. (2023). Discoid lateral meniscus instability in children: Part I. A new grading system of instability to clarify natural history. Knee Surg Sports Traumatol Arthrosc.

[32] Niu E.L., Kinnard M.J., Hoyt B.W., Zember J., Murphy T.P. (2024). Magnetic resonance imaging indirect signs for anterior instability of the lateral meniscus in pediatric and adolescent patients. J Pediatr Orthop.

[33] Kluczynski M.A., Marzo J.M., Rauh M.A., Bernas G.A., Bisson L.J. (2015). Sex-specific predictors of intra-articular injuries observed during anterior cruciate ligament reconstruction. Orthop J Sports Med.

[34] Yoo W.J., Choi I.H., Chung C.Y. (2008). Discoid lateral meniscus in children: Limited knee extension and meniscal instability in the posterior segment. J Pediatr Orthop.

[35] Ahn J.H., Lee Y.S., Ha H.C., Shim J.S., Lim K.S. (2009). A novel magnetic resonance imaging classification of discoid lateral meniscus based on peripheral attachment. Am J Sports Med.

[36] Jin B., Zhen J., Wei X. (2021). Evaluation of the peripheral rim instability of the discoid meniscus in children by using weight-bearing magnetic resonance imaging. J Comput Assist Tomogr.

[37] Campbell A.L., Pace J.L., Mandelbaum B.R. (2023). Discoid lateral meniscus. Curr Rev Musculoskelet Med.

[38] Bauwens P.H., Vandergugten S., Fiquet C., Raux S., Cance N., Chotel F. (2023). Discoid lateral meniscus instability in children: Part II. Repair first to minimise the saucerisation. Knee Surg Sports Traumatol Arthrosc.

[39] Talathi N., Bennett A., Chiou D., Beck J. (2024). Patient-reported outcomes after surgically treated anterior horn tears in the pediatric discoid meniscus. Orthop J Sports Med.

